# Refeeding-Syndrom: Pathophysiologie, Therapie und welche rheumatologischen Patienten besonders gefährdet sind

**DOI:** 10.1007/s00393-020-00952-7

**Published:** 2020-12-23

**Authors:** P. Nguyen, H. Schlögl, L. Selig, C. Baerwald

**Affiliations:** 1 Klinik und Poliklinik für Endokrinologie, Nephrologie, Rheumatologie, Bereich Rheumatologie, Universitätsklinikum Leipzig, Liebigstr. 20, 04103 Leipzig, Deutschland; 2 Klinik und Poliklinik für Endokrinologie, Nephrologie, Rheumatologie, Bereich Endokrinologie und Spezialbereich Ernährungsmedizin, Universitätsklinikum Leipzig, Leipzig, Deutschland

**Keywords:** Refeeding-Syndrom, Mangelernährung, Hypophosphatämie, Ernährungstherapie, Rheumatische Krankheiten, Refeeding syndrome, Malnutrition, Hypophosphatemia, Nutritional therapy, Rheumatic diseases

## Abstract

Rheumatische Krankheiten können über verschiedene Mechanismen zu einer Mangelernährung, also einer nicht ausreichenden Deckung des Bedarfs an Energie, Proteinen, Elektrolyten oder anderen Nährstoffen, führen. Bei Wiederbeginn mit vollwertiger Ernährung nach einer Phase einer solchen katabolen Stoffwechsellage kommt es zu metabolischen Veränderungen, die in einen akuten Mangel an verfügbaren Elektrolyten und anderen Mikronährstoffen führen und dann lebensbedrohliche Komplikationen auslösen können. Das Auftreten solcher Komplikationen nach Wiederbeginn der Ernährung wird als Refeeding-Syndrom bezeichnet. Mit Wissen um diese Komplikationen, dem adäquaten Wiederbeginn der Ernährung und ggf. einer Überwachung der relevanten Parameter sowie gezielter Supplementierung kann das Refeeding-Syndrom vermieden werden. In dieser Übersichtsarbeit werden die Pathomechanismen des Refeeding-Syndroms erklärt, die Risikofaktoren für das Auftreten des Refeeding-Syndroms – insbesondere unter Betrachtung von rheumatologischen Patienten – identifiziert und die nötige Therapie zur Vermeidung eines Refeeding-Syndroms bei Wiederbeginn der Ernährung dargestellt.

## Abstract (en)

Rheumatic diseases can lead to a state of malnutrition via a variety of mechanisms. Malnutrition is defined as an insufficient availability of energy, proteins, electrolytes and other nutrients compared to the requirements of a healthy body. After such a catabolic phase, a sudden resupply of the body’s full caloric needs can cause life-threatening complications due to an acute paucity of electrolytes and micronutrients. Such metabolic disturbances occurring after the reconstitution of nutrition are termed refeeding syndrome. With sufficient background knowledge about the refeeding syndrome, physicians can prevent serious complications for patients through an adequate reconstitution of caloric intake, the monitoring of relevant laboratory parameters and the supplementation of deficient electrolytes and micronutrients. This review aims to explain the pathological mechanisms driving the refeeding syndrome, to identify risk factors for developing a refeeding syndrome especially in patients with rheumatic diseases and to present strategies to prevent the occurrence of the refeeding syndrome during nutrient reconstitution.

## Hintergrund

Die Ernährung spielt eine wichtige Rolle im Krankheitsmanagement von Patienten mit chronischen Krankheiten. Auch in der Rheumatologie wird eine Abweichung vom optimalen Ernährungsstatus, insbesondere im Sinne einer Mangelernährung, häufig unterschätzt und ist mit einem schlechteren Krankheitsverlauf assoziiert [10]. Bei mangelernährten Patienten stellt bei Wiederaufnahme der Ernährung das Refeeding-Syndrom eine zusätzliche Gefahr dar. Diese Komplikation ist durch potenziell lebensbedrohliche Elektrolytstörungen gekennzeichnet, die auf dem Boden einer zu schnellen Nährstoffzufuhr bei vorbestehendem Hungerstoffwechsel entstehen. Dieser Artikel erläutert für praktisch tätige Rheumatologen die Pathophysiologie des Refeeding-Syndroms, identifiziert relevante Gruppen von Risikopatienten und erläutert Präventionsmaßnahmen sowie therapeutische Herangehensweisen nach aktuellem Stand der Empfehlungen.

## Definition des Refeeding-Syndroms

Das Refeeding-Syndrom wird als eine schwere Elektrolyt- und Mikronährstoffstörung bei mangelernährten Patienten definiert, die nach zu raschem Beginn einer Ernährungstherapie eintritt [23]. Das lange Fehlen von standardisierten diagnostischen Kriterien führte zu einer erschwerten Erfassung des Erkrankungsbildes und zur schlechten Vergleichbarkeit von wissenschaftlichen Untersuchungen. Um dem entgegenzuwirken, veröffentlichte die Amerikanische Gesellschaft für parenterale und enterale Ernährung (American Society for Parenteral and Enteral Nutrition [ASPEN]) 2020 die ersten Konsensuskriterien für die Diagnosestellung eines Refeeding-Syndroms ([22]; Tab. 1).

**Table 1:** 

**Diagnose Refeeding-Syndrom**: kann bei Erfüllung beider Kriterien gestellt werden:	
**1. Kriterium:** *Abfall der Serumkonzentration* **eines oder mehrerer** *der folgenden Elektrolyte:* Phosphat Kalium Magnesium	*Schweregradeinteilung:* Mild: ↓ 10–20 % Moderat: ↓ 20–30 % Schwer: ↓ >30 % *oder* zusätzlich Thiaminmangel
**2. Kriterium:** Eintritt innerhalb von **5 Tagen** nach Beginn einer erhöhten Energiezufuhr	

*ASPEN* American Society for Parenteral and Enteral Nutrition

Für die Diagnose ist nach der ASPEN die Schwere der Störung des Phosphat‑, Kalium- und/oder Magnesiumhaushaltes ausschlaggebend, wobei ein mindestens 10 %iges Absinken der Serumkonzentration des jeweiligen Elektrolyts unter den Normbereich innerhalb der ersten 5 Tage nach Wiederbeginn der Energiezufuhr auftritt [6]. Anhand der prozentualen Abweichung vom Normwert kann der Schweregrad eines Refeeding-Syndroms in mild (Elektrolyt 10–20 % unterhalb der Norm), moderat (20–30 % darunter) oder schwer (>30 % unter der Norm) eingeteilt werden. Ein Thiaminmangel in Kombination mit einer Elektrolytstörung jeden Ausmaßes reicht aus, um ein Refeeding-Syndrom als schwer einzustufen.

Der schnelle und potenziell schwerwiegende Verlauf macht das Refeeding-Syndrom zu einer gefährlichen Komplikation für Risikopatienten. Bis zu 8 % der internistischen Patienten und 14 % der Patienten mit einer vorbestehenden mangelhaften Ernährung entwickeln im stationären Verlauf ein Refeeding-Syndrom [10, 15]. Diese Patienten haben eine 1,5-fach erhöhte Mortalität, sowie ein deutlich erhöhtes Risiko für eine Intensivverlegung und einen protrahierten Krankenhausaufenthalt im Vergleich zu Krankenhauspatienten ohne Refeeding-Syndrom [10].

## Pathophysiologie

Während einer Periode mit fehlender oder ungenügender Nährstoffzufuhr passt sich der Stoffwechsel des menschlichen Körpers der Mangelsituation an und stellt von der Energiegewinnung durch Kohlenhydrate auf die Energiegewinnung durch Verbrennen von Proteinen und Fetten um. Zu Beginn des Nährstoffmangels wird noch Glukose aus in der Leber gespeichertem Glykogen freigesetzt. Dieser Vorrat ist nach ca. 12–24 h erschöpft.

Danach stellt der Körper auf andere Mechanismen zur Energiegewinnung um: über die Glukoneogenese in der Leber kann auch nach Erschöpfung der Glykogenspeicher weiter Glukose zur Verfügung gestellt werden. Als Substrate für die Glukosebildung dienen dann Ketone, Glycerin und Aminosäuren, die aus Fettgewebe und Muskulatur freigesetzt werden. Außerdem können freie Fettsäuren in der Leber in Ketone umgewandelt werden, die als direkte Energiequelle in vielen Geweben, einschließlich des Gehirns, dienen können [17]. So kommt es durch den Abbau von Fettgewebe und Muskulatur zum Verlust von Zellmasse und damit zum Verlust von intrazellulär gespeichertem Phosphat, Magnesium und Kalium. Die Serumkonzentrationen dieser Elektrolyte bleiben zunächst unbeeinflusst. Der damit aus der Mangelernährung resultierende Muskelabbau kann auch das Myokard betreffen und zu einer Abnahme der Herzmuskelkontraktilität führen [25]. Wenn nun nach der Fastenperiode die Versorgung mit exogenen Kohlenhydraten plötzlich wiederhergestellt wird, stellt der Organismus schnell wieder auf den vor Beginn der Mangelperiode bestehenden anabolen Stoffwechsel mit hohem Substratbedarf um, was aufgrund des aber mittlerweile bestehenden Mangels an Elektrolyten und anderer Mikronährstoffe zu schwerwiegenden Komplikationen führen kann, die im Folgenden erläutert werden.

**Figure 1:**
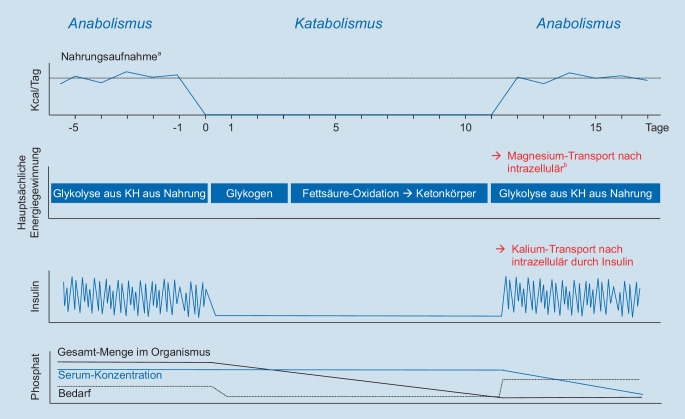

Nach Kohlenhydratzufuhr steigt die Insulinkonzentration schnell an (Abb. 1), und die aufgenommene Glukose wird in der Glykolyse wieder zu Pyruvat abgebaut. Dabei tritt aufgrund der Bildung von Adenosintriphosphat (ATP) ein hoher Phosphatverbrauch auf. Dies kann dann in einer Hypophosphatämie resultieren, die zu einer verminderten Kontraktilität von glatten und quergestreiften Muskeln (einschließlich Herz- und Atemmuskulatur) führen kann. Durch das Insulin kommt es außerdem durch die Aktivierung des Glukosetransporters zusammen mit Glukose zu einem Transport von Kalium und Magnesium nach intrazellulär. Magnesium gelangt außerdem als Folge des erhöhten Verbrauchs als Koenzym bei verschiedenen intrazellulären metabolischen Prozessen in die Zelle. Die Folge sind niedrigere Serumkonzentrationen dieser beiden Elektrolyte. Hauptkomplikationen davon sind neuromuskuläre Störungen und Herzrhythmusstörungen [27]. Eine hohe Insulinkonzentration im Blut stimuliert außerdem die Natrium- und Wasserretention. In Kombination mit einer durch die oben beschriebenen Mechanismen eingeschränkten kardialen Pumpfunktion kann dies zu einer kardialen Dekompensation führen.

Da nach Wiederbeginn der (kohlenhydratreichen) Ernährung nun wieder Substrat für die Glykolyse vorhanden ist, wird auch wieder Pyruvat für den Citratzyklus bereitgestellt und dabei Thiamin (Synonym: Vitamin B_1_) verbraucht, das als Koenzym fungiert. Da die Speicherkapazität für Thiamin im menschlichen Körper begrenzt ist, kommt es schnell zu einem Mangel, der bei stärkerer Ausprägung zu einer Wernicke-Enzephalopathie oder Beriberi führen kann [8]. Ohne Thiamin kann das in der Glykolyse gebildete Pyruvat nicht in den Citratzyklus gelangen und wird zu Laktat umgewandelt, was zu einer Laktatazidose führen kann.

## Gefährdete Patienten in der Rheumatologie

Der größte Risikofaktor für die Entstehung eines Refeeding-Syndroms ist eine vorbestehende Mangelernährung. Demzufolge sind Patienten mit konsumierenden Erkrankungen, wie z. B. Patienten mit malignen Erkrankungen, besonders gefährdet. Außerdem zählen zu der Risikokohorte Patienten mit einer chronischen Herz- oder Lungenerkrankung, aber auch Patienten mit schwerwiegenden psychischen Erkrankungen wie *Anorexia nervosa* oder chronischem Alkoholabusus [15, 24]. Oft übersehen werden zudem Patienten mit chronischen Infektionen und akutgeriatrische Patienten, bei welchen zu ca. 50 % eine Mangelernährung besteht [7, 10].

Daten zur Inzidenz des Refeeding-Syndroms bei Patienten mit rheumatischen Krankheiten sind lückenhaft. Aus den publizierten Fallberichten lässt sich jedoch ein vorbestehender pathologischer Ernährungsstatus in allen Kasuistiken erfassen [12, 26]. Im Folgenden werden entzündlich rheumatische Krankheiten und die möglichen Mechanismen diskutiert, die die Patienten einem erhöhten Risiko für eine Mangelernährung und somit bei Wiederaufnahme einer adäquaten Ernährung der Entwicklung eines Refeeding-Syndroms aussetzen.

Eine Mangelernährung kann bei allen rheumatischen Krankheiten vorkommen. Die höchste Prävalenz findet sich bei Patienten mit einer systemischen Sklerose. Wird die (quantitative) Mangelernährung hauptsächlich anhand des Body-Mass-Indexes (BMI) oder eines raschen Gewichtsverlusts (≥10 % innerhalb von 6 Monaten) festgestellt, finden sich Prävalenzen um 15 % [4]. Werden technisch höherwertige Messungen, wie z. B. die Bioimpedanzmessung, durchgeführt, mit der näherungsweise zwischen Körperfett und Muskelmasse differenziert werden kann, zeigen sich bei Patienten mit systemischer Sklerose Prävalenzen bis zu 55 %, die somit die klinisch deutliche Unterschätzung der Mangelernährung aufdecken [16].

Ein Faktor für das Auftreten einer Mangelernährung bei systemischer Sklerose scheint das Ausmaß der pulmonalen Beteiligung zu sein. In Beobachtungsstudien stellte eine reduzierte funktionale Vitalkapazität bei Patienten mit systemischer Sklerose und interstitieller Lungenerkrankung einen wichtigen Risikofaktor für das Auftreten einer Mangelernährung dar [3, 4, 16]. Als zugrunde liegender Mechanismus können die erhöhte Atemmuskelarbeit und damit der erhöhte Energieverbrauch vermutet werden; eine gute Evidenz dafür fehlt jedoch bisher. Ein insgesamt schwerer Krankheitsverlauf, der meist mit einer Lungenbeteiligung einhergeht, kann als Störfaktor in den großen retrospektiven Analysen eine Rolle gespielt haben. Interessanterweise hat sich entgegen den Vermutungen keine statistisch signifikante Assoziation zwischen einer gastrointestinalen Beteiligung und der Mangelernährung ergeben [3, 4, 16]. Basierend auf pathophysiologischen Überlegungen sollte dennoch auf die Mangelernährung bei Patienten mit gastrointestinaler Motilitätsstörung geachtet werden, insbesondere mit den häufigen Symptomen der Übelkeit, des Erbrechens und des Völlegefühls. Im fortgeschrittenen Stadium stellen die Dysphagie und Mikrostomie oft erhebliche mechanische Barrieren für die Nahrungsaufnahme dar. Auch die bekannte Vaskulopathie der Darmschleimhaut und die damit einhergehende Malabsorption können das Risiko einer Mangelernährung verstärken [22].

Eine relevante Darmbeteiligung findet sich ebenfalls bei Patienten mit einer Spondyloarthritis und den damit assoziierten chronisch entzündlichen Darmerkrankungen. Auch wenn keine Untersuchungen an primär rheumatologischen Patienten vorliegen, konnte bei Patienten mit einem Morbus Crohn oder einer Colitis ulcerosa in 16 % der Fälle eine Mangelernährung registriert werden [5]. Eine hohe Krankheitsaktivität wurde als wichtigster Risikofaktor identifiziert, der das Risiko einer Mangelernährung um das 4‑Fache erhöhte. In Anbetracht der in der Rheumatologie durchaus präsenten Patientengruppe mit chronisch entzündlichen Darmerkrankungen als Komorbidität sollte auch hier für eine umfassende Patientenbetreuung der Ernährungsstatus im Auge behalten werden.

Eine Nierenbeteiligung ist im Rahmen einer Lupusnephritis oder Vaskulitis ebenfalls mit einem erhöhten Risiko für die Mangelernährung assoziiert [20]. Besonders Dialysepatienten sind durch einen gesteigerten Katabolismus aufgrund der chronischen Azidose, dem gestörten Hormonhaushalt und den dialysebedingten, persistierenden proinflammatorischen Signalen im Blut gefährdet. Zudem lässt sich trotz moderner Dialysetechniken der Nährstoffverlust über die Dialyse noch nicht ausreichend reduzieren [2]. Die Bedeutung dieser Mechanismen zeigt sich in der hohen Prävalenz der Mangelernährung mit bis zu 44 % unter den dialysepflichtigen ANCA-assoziierten Vaskulitispatienten [20].

Bei der in der Rheumatologie deutlich häufiger vorkommenden rheumatoiden Arthritis zeigte sich in mehreren Querschnittstudien eine Prävalenz der Mangelernährung von ca. 25 % [11, 13, 14]. Der schlechte Ernährungsstatus korreliert auch bei dieser Erkrankung mit der Krankheitsaktivität [21], insbesondere mit den laborchemischen Parametern Blutsenkungsgeschwindigkeit, CRP [13], aber auch mit einer Hypalbuminämie [11]. In einer prospektiven Studie konnte die hohe Krankheitsaktivität als wichtigster Risikofaktor für die Reduktion der Muskelmasse bei Patienten mit rheumatoider Arthritis festgestellt werden [21]. Als Pathomechanismus lässt sich vermuten, dass die aktive Entzündung den katabolen Stoffwechsel unterhält und auf Dauer eine Mangelernährung begünstigt. Weiterhin können Schmerzen, Fatigue, generelles Unwohlsein und der damit einhergehende Appetitverlust bei Patienten mit häufigen Schüben das Risiko für die Mangelernährung erhöhen. Diese genannten Mechanismen lassen sich ebenfalls für Erkrankungen mit einer hohen basalen Entzündungsaktivität und häufigen Schüben wie Riesenzellarteriitis oder Takayasu-Arteriitis vermuten.

**Figure 2:**
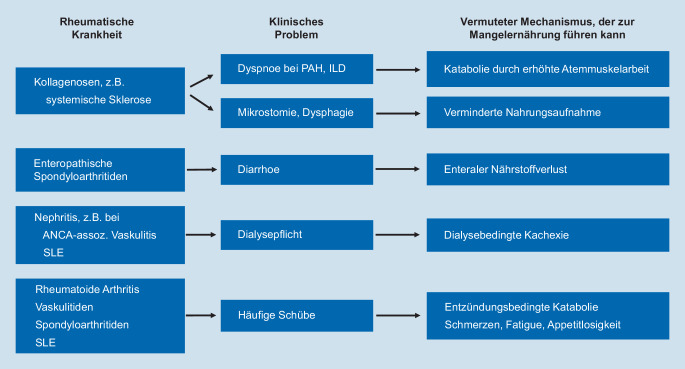

Die Abb. 2 veranschaulicht die häufigen Symptome und die damit assoziierten rheumatischen Krankheiten, bei denen ein besonderes Augenmerk auf den Ernährungsstatus der Patienten gerichtet werden soll. Eine Mangelernährung sollte frühzeitig erkannt und angemessen behoben werden, um das potenziell tödliche Refeeding-Syndrom zu vermeiden.

## Management des Refeeding-Syndroms

Es ist das primäre Ziel, bei mangelernährten Patienten, bei denen eine Ernährung wieder begonnen wird, ein Refeeding-Syndrom durch die entsprechenden Maßnahmen zu verhindern. Dafür ist das frühzeitige Identifizieren der Risikopatienten essenziell. Hierfür wurden diverse Scoring-Systeme geschaffen, worunter sich in den europäischen Leitlinien die britischen Leitlinien des *National Institute for Health and Care Excellence* (NICE) durchsetzen konnten (Tab. 2). Sowohl ein BMI <16 kg/m^2^ als auch ein Gewichtsverlust von >15 % in dem letzten halben Jahr sind alleinstehend Kriterien für ein erhöhtes Refeeding-Syndrom-Risiko. Genauso risikobehaftet sind Patienten mit einem guten Ernährungsstatus, die jedoch z. B. aufgrund eines starken Krankheitsschubes in den letzten 10 Tagen kaum Nahrung zu sich nehmen konnten. Zeigen sie ohne eine andere erkennbare Ursache basal niedrige Serumkonzentrationen von Phosphat, Kalium und/oder Magnesium, zählen auch diese Patienten zur Risikogruppe. Außerdem liegt ein erhöhtes Risiko für ein Refeeding-Syndrom vor, wenn mindestens 2 der folgenden Kriterien erfüllt sind: BMI <18,5 kg/m^2^, ungewollter Gewichtsverlust von >10 % in den letzten 6 Monaten, unzureichende Nahrungszufuhr in den letzten 5 Tagen, chronischer Alkoholabusus, Medikation mit Insulin, Chemotherapeutika, Antazida oder Diuretika (Tab. 2; [28]).

**Table 2:** 

**Es besteht ein erhöhtes Risiko für das Auftreten eines Refeeding-Syndroms, wenn der Patient von den folgenden Kriterien:**		
**Mindestens 1 Kriterium erfüllt:**	**ODER**	**Mindestens 2 Kriterien erfüllt:**
BMI <16 kg/m^2^		BMI <18,5 kg/m^2^
Ungewollter Gewichtsverlust >15 % in den letzten 6 Monaten		Ungewollter Gewichtsverlust >10 % in den letzten 6 Monaten
Kaum oder keine Nahrungszunahme in den letzten 10 Tagen		Kaum oder keine Nahrungszunahme in den letzten 5 Tagen
Niedrige Serumkonzentration an Phosphat, Kalium und/oder Magnesium		In der Vorgeschichte chronischer Alkoholabusus oder Medikation mit Insulin, Chemotherapeutika, Antazida oder Diuretika

*BMI* Body-Mass-Index, *NICE* National Institute for Health and Care Excellence

**Figure 3:**
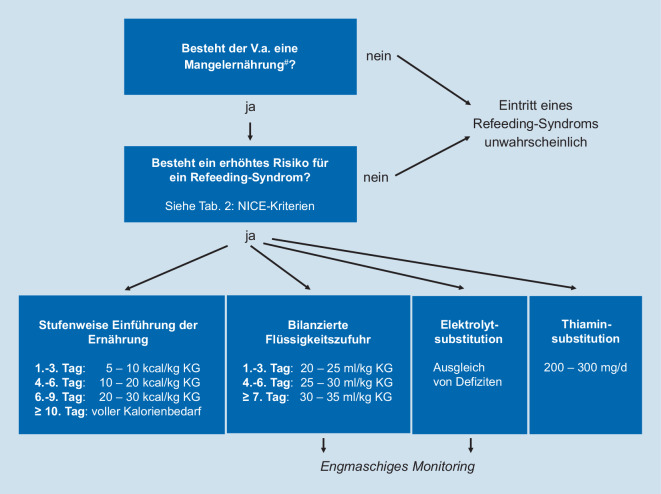

Die ernährungstherapeutische Intervention zum Wiederbeginn der Ernährung besteht aus einer verringerten Energiezufuhr im Verhältnis zum theoretisch berechneten Energiebedarf und in der ausreichenden Deckung des anfänglich immer erhöhten Bedarfs an Elektrolyten. Auch wenn sich in den bisherigen Studien kein Protokoll zur Ernährungsinitiierung durchsetzen konnte, akkumuliert die Evidenz zugunsten einer restriktiven Kalorienzufuhr. Eine randomisiert kontrollierte Studie konnte für die restriktive Kalorienzufuhr eine deutlich verringerte Mortalität nachweisen [9] und dadurch die Ergebnisse von retrospektiven Studien bekräftigen [19]. Dieses Resultat spiegelte sich in den Empfehlungen verschiedener Fachgesellschaften und Expertengruppen wider [1, 22]. Die gängige Praxis in Deutschland besteht aus einer stufenweisen Einführung der Ernährung: am ersten bis dritten Tag der Wiedereinführung der Ernährung 5–10 kcal/kg Körpergewicht, am vierten bis sechsten Tag 10–20 kcal/kg Körpergewicht, am siebten bis neunten Tag 20–30 kcal/Tag und ab dem 10. Tag der volle Kalorienbedarf (Abb. 3; [1]).

Zeitgleich mit dem Wiederbeginn der Ernährung sollte neben der Korrektur von Elektrolyt- und Flüssigkeitsverschiebungen eine Thiaminsubstitution erfolgen. Eine Korrektur von Elektrolyt- und Flüssigkeitsverschiebungen sollte ebenfalls zeitgleich mit der Ernährung erfolgen [18]. Empfehlungen für die Substitutionsrate und die Monitoringintervalle je nach Schweregrad des Elektrolytmangels finden sich in Tab. 3. Um eine Überladung mit Flüssigkeit zu vermeiden, empfiehlt sich eine Flüssigkeitszufuhr vom ersten bis zum dritten Tag von 20–25 ml/kg Körpergewicht, vom vierten bis sechsten Tag von 25–30 ml/kg Körpergewicht und ab dem siebten Tag von 30–35 ml/kg Körpergewicht (Abb. 3; [1]). Bei Patienten mit kardialer Vorschädigung und dadurch erhöhtem Risiko einer kardialen Dekompensation sollten Blutdruck und Puls mehrmals täglich kontrolliert werden. Bei der Wahl der Flüssigkeit sind nach aktuellem Wissensstand balancierte Elektrolytlösungen, wie z. B. Ringer-Acetat, gegenüber NaCl 0,9 % empfohlen. Die Natriumgabe sollte sich auf den Ersatz von Verlusten beschränken.

**Table 3:** 

	Kalium	Magnesium	Phosphat
Milder Mangel	*Serumkonzentration:* 3,1–3,5 mmol/l	0,5–0,6 mmol/l	0,61–0,8 mmol/l
	20 mmol/l oral oder *i.v.* über 4–8 h Kontrolle nach 24 h	10–15 mmol/l oder geteilte Dosen von 5–10 mmol/l (um Diarrhö zu vermeiden)	0,3 mmol/kg/Tag oral oder *i.v.* über 8–12 h Kontrolle nach 24 h
Moderater Mangel	*Serumkonzentration:* 2,5–3,0 mmol/l	Wie milder Mangel	0,3–0,6 mmol/l
	20–40 mmol/l über 4–8 h Kontrolle nach 8 h, wenn nicht normal, mit 20 mmol/l weiter	Gleiches Vorgehen wie beim milden Mangel	0,6 mmol/kg/Tag über 8–12 h, maximal 50 mmol in 24 h Kontrolle nach 8–12 h, Wiederholung wenn nötig
Schwerer Mangel	*Serumkonzentration:* <2,5 mmol/l	<0,5 mmol/l	<0,3 mmol/l
	40 mmol/l *i.v.* über 4–8 h Kontrolle nach 8 h, wenn nicht normal, mit 40 mmol/l weiter	20–24 mmol/l (4–6 g) *i.v.* über 4–8 h Kontrolle jeweils nach 8–12 h	Gleiches Vorgehen wie beim moderaten Mangel

### Infobox 1 Exkurs in die Ernährungsmedizin

- Mangelernährung: Sammelbegriff für Ernährungszustände, in denen der Bedarf an Energieträgern (Kohlenhydrate, Fette, Proteine) und/oder anderen Nährstoffen wie Elektrolyte, Vitamine etc. nicht gedeckt ist. Die krankheitsassoziierten Formen teilt die Deutsche Gesellschaft für Ernährungsmedizin (DGEM) in ihren Leitlinien von 2013 ein in: akute (z. B. im Rahmen einer Sepsis) und die chronische (z. B. bei gastrointestinalen Resorptionsproblemen bei chronisch entzündlichen Darmerkrankungen) Form [23]. Mangelernährung ist unabhängig vom Gewicht, denn auch adipöse Menschen, die sich z. B. sehr einseitig ernähren oder aufgrund einer bariatrischen Operation eine Malabsorption haben, können einen Mangel an bestimmten Vitaminen oder Mineralstoffen haben und damit qualitativ mangelernährt sein
- Unterernährung: nicht ausreichende Deckung des Energiebedarfs durch Makronährstoffe, die dann zum Untergewicht führt und meist mit einem Eiweiß‑, Fett‑, Vitamin- und Mineralstoffmangel einhergeht
- Makronährstoffe: die zur Energiegewinnung dienenden Nährstoffe Fette, Kohlenhydrate und Proteine
- Mikronährstoffe: alle für den Körper essenziellen Nährstoffe, die nicht der Energiegewinnung dienen: u. a. Vitamine und Mineralstoffe (dazu gehören die sog. Mengenelemente wie Kalium, Natrium, Calcium, Phosphat und die Spurenelemente wie Eisen, Jod, Zink)
- Thiamin (Synonym: Vitamin B_1_): Vitamin, das u. a. in Vollkorn, Hülsenfrüchten und einigen Fleisch- und Fischsorten vorkommt. Dient u. a. als Koenzym bei der Umwandlung von Pyruvat zu Acetyl-CoA in den Mitochondrien. Ein Thiaminmangel betrifft v. a. Zellen und Organe mit ausgeprägtem Glukosestoffwechsel (u. a. Muskel- und Nervenzellen) und kann schwere Erkrankungen wie die Wernicke-Enzephalopathie und Beriberi auslösen
- Anabolismus: die (Energie verbrauchenden) metabolischen Prozesse, die größere Moleküle aus kleineren Einheiten bilden und so zum Aufbau von Körpermasse dienen
- Katabolismus: Abbau von komplexen Molekülen (wie Proteinen, Polysacchariden, Lipiden) in kleinere Einheiten (wie Aminosäuren, Monosacchariden, Fettsäuren) entweder durch Oxidation zur Energiegewinnung oder als Substrate für anabole Reaktionen

## Fazit für die Praxis

- Das Refeeding-Syndrom ist eine potenziell lebensbedrohliche Komplikation infolge einer zu schnellen Wiederaufnahme der Ernährung im Mangelzustand.
- Im Vordergrund der Pathogenese steht ein akuter Mangel an Phosphat, Kalium, Magnesium und Thiamin mit entsprechenden klinischen Zeichen einer neuromuskulären, kardialen und/oder zentralnervösen Störung.
- Essenziell für die Prävention eines Refeeding-Syndroms sind das frühzeitige Erkennen der Risikopatienten und eine restriktive Kalorienzufuhr mit entsprechendem Monitoring des Elektrolythaushaltes.
- In der Rheumatologie sind v. a. Patienten gefährdet, die wegen der Grunderkrankung eine Mangelernährung aufweisen. Ist bei solchen Patienten eine Erhöhung der Energiezufuhr geplant, muss auf einen möglichen Eintritt eines Refeeding-Syndroms besonders geachtet werden.

## References

[1] Aubry E, Aeberhard C, Leuenberger M (2019). Refeeding-Syndrom: Ein konsensusbasierter Algorithmus für stationäre Patienten. Aktuel Ernahrungsmed.

[2] Bossola M, Muscaritoli M, Tazza L (2005). Malnutrition in hemodialysis patients: what therapy?. Am J Kidney Dis.

[3] Caimmi C, Caramaschi P, Venturini A (2018). Malnutrition and sarcopenia in a large cohort of patients with systemic sclerosis. Clin Rheumatol.

[4] Caporali R, Caccialanza R, Bonino C (2012). Disease-related malnutrition in outpatients with systemic sclerosis. Clin Nutr.

[5] Casanova MJ, Chaparro M, Molina B (2017). Prevalence of malnutrition and nutritional characteristics of patients with inflammatory bowel disease. J Crohns Colitis.

[6] Cederholm T, Barazzoni R, Austin P (2017). ESPEN guidelines on definitions and terminology of clinical nutrition. Clin Nutr.

[7] Cerri AP, Bellelli G, Mazzone A (2015). Sarcopenia and malnutrition in acutely ill hospitalized elderly: prevalence and outcomes. Clin Nutr.

[8] Crook MA (2014). Refeeding syndrome: problems with definition and management. Nutrition.

[9] Doig GS, Simpson F, Heighes PT (2015). Restricted versus continued standard caloric intake during the management of refeeding syndrome in critically ill adults: a randomised, parallel-group, multicentre, single-blind controlled trial. Lancet Respir Med.

[10] Friedli N, Baumann J, Hummel R (2020). Refeeding syndrome is associated with increased mortality in malnourished medical inpatients: secondary analysis of a randomized trial. Medicine (Baltimore).

[11] Fukuda W, Yamazaki T, Akaogi T (2005). Malnutrition and disease progression in patients with rheumatoid arthritis. Mod Rheumatol.

[12] Gordon M, Riley P (2011). Refeeding syndrome in a child with juvenile dermatomyositis. Arch Dis Child.

[13] Helliwell M, Coombes EJ, Moody BJ (1984). Nutritional status in patients with rheumatoid arthritis. Ann Rheum Dis.

[14] Hernandez-beriain JA, Segura-garcia C, Rodriguez-lozano B (1996). Undernutrition in rheumatoid arthritis patients with disability. Scand J Rheumatol.

[15] Kraaijenbrink BVC, Lambers WM, Mathus-Vliegen EMH, Siegert CEH (2016). Incidence of refeeding syndrome in internal medicine patients. Neth J Med.

[16] Krause L, Becker MO, Brueckner CS (2010). Nutritional status as marker for disease activity and severity predicting mortality in patients with systemic sclerosis. Ann Rheum Dis.

[17] Longo VD, Mattson MP (2014). Fasting: molecular mechanisms and clinical applications. Cell Metab.

[18] Mehanna HM, Moledina J, Travis J (2008). Refeeding syndrome: what it is, and how to prevent and treat it. BMJ.

[19] Olthof LE, Koekkoek WACK, van Setten C (2018). Impact of caloric intake in critically ill patients with, and without, refeeding syndrome: a retrospective study. Clin Nutr.

[20] Romeu M, Couchoud C, Delarozière J-C (2014). Survival of patients with ANCA-associated vasculitis on chronic dialysis: data from the French REIN registry from 2002 to 2011. QJM.

[21] Santos F, Borges MC, Correia MITD (2010) Assessment of nutritional status and physical activity in systemic lupus erythematosus patients. Bras J Rheumatol 50(6):631–638 21243304

[22] da Silva JSV, Seres DS, Sabino K (2020). ASPEN consensus recommendations for refeeding syndrome. Nutr Clin Pract.

[23] Valentini L, Volkert D, Schütz T (2013). Leitlinie der Deutschen Gesellschaft für Ernährungsmedizin (DGEM). Aktuel Ernahrungsmed.

[24] Walmsley RS (2013). Refeeding syndrome: screening, incidence, and treatment during parenteral nutrition. J Gastroenterol Hepatol.

[25] Weinsier RL, Krumdieck CL (1981). Death resulting from overzealous total parenteral nutrition: the refeeding syndrome revisited. Am J Clin Nutr.

[26] Windpessl M, Mayrbaeurl B, Baldinger C (2017). Refeeding syndrome in oncology: report of four cases. World J Oncol.

[27] Wirth R, Diekmann R, Janssen G (2018). Refeeding-Syndrom: Pathophysiologie, Risikofaktoren, Prophylaxe und Therapie. Internist.

[28] NICE (2017) Nutrition support for adults: oral nutrition support, enteral tube feeding and parenteral nutrition. https://www.nice.org.uk/guidance/cg32. Zugegriffen: 3. Okt. 2020 31999417

